# Comparative analysis of small extracellular vesicles in peritoneal fluid from endometriosis and non-endometriosis patients: a pilot case-control study

**DOI:** 10.20517/evcna.2026.23

**Published:** 2026-07-22

**Authors:** Barbara Bortot, Alice Gualerzi, Roberta Di Florio, Silvia Picciolini, Aurora Mangolini, Francesco Valle, Marco Brucale, Federico Romano, Giuseppe Ricci, Stefania Biffi

**Affiliations:** ^1^Institute for Maternal and Child Health, IRCCS Burlo Garofolo, Trieste 34137, Italy.; ^2^IRCCS Fondazione Don Carlo Gnocchi, Milan 20148, Italy.; ^3^Institute of Nanostructured Materials, Italian National Council of Research, Bologna 40129, Italy.; ^4^Department of Medical, Surgical and Health Science, University of Trieste, Trieste 34129, Italy.

**Keywords:** Endometriosis, peritoneal fluid, extracellular vesicles, Raman spectroscopy, single-particle interferometric reflectance imaging sensing, atomic force microscopy

## Abstract

**Aim:** Endometriosis is a chronic inflammatory disorder requiring clinical examination, imaging, and surgical intervention for accurate assessment. The aim of the present study was to provide a detailed biophysical and biochemical characterization of small extracellular vesicles (sEVs) derived from peritoneal fluid, and to explore disease-associated molecular differences that may inform future investigations.

**Methods:** sEVs were isolated from the peritoneal fluid of women with endometriosis (*n* = 7), women without endometriosis who underwent surgery for benign, noninflammatory conditions (*n* = 7), and follicular fluid samples (*n* = 3). The tetraspanin profile of the samples was determined using single-particle interferometric reflectance imaging sensing. After size exclusion chromatography and ultracentrifugation, atomic force microscopy (AFM) and Raman spectroscopy were employed to characterize the sEVs.

**Results:** Prominent Raman spectral variations in peritoneal sEVs between control women and those with endometriosis were observed at 891 cm^-1^ (proteins) and 968 cm^-1^ (lipids, CH wagging). Statistical analysis of molecular components influencing the Raman signature of sEVs suggest that their lipid composition appears to be influenced by the pathophysiology of endometriosis, reflecting changes similar to those observed in the eutopic endometrium in previous studies. sEVs isolated from peritoneal fluid and follicular fluid have markedly distinct biochemical compositions and morphometric properties. sEVs from follicular fluid were significantly smaller in size and exhibited a higher rigidity in this pilot cohort.

**Conclusion:** Distinct biophysical and biochemical differences in sEVs from endometriosis patients are documented in this pilot study, contributing to a deeper characterization of disease-associated extracellular vesicle (EV) alterations. Variations between follicular and peritoneal fluid sEVs suggest unique functional roles in reproductive health. These findings are hypothesis-generating and warrant validation in larger, well-controlled cohorts before any diagnostic application can be claimed.

## INTRODUCTION

Endometriosis is a chronic inflammatory condition associated with an increased risk of infertility^[1,2]^. The condition is characterized by the presence of endometrial-like glands and stroma in areas beyond the uterine cavity, potentially presenting with dysmenorrhea, infertility, chronic pelvic pain, and a variety of other symptoms, with little association between the extent of the disease and the severity of symptoms^[1,2]^. The current diagnosis of endometriosis relies on clinical history and examination, ultrasound, and finally, laparoscopic surgery and biopsy^[3]^. Although endometriomas and deep infiltrating endometriosis may be detected with greater accuracy with the use of ultrasound, the most common form of superficial peritoneal disease cannot be accurately predicted without a surgical procedure^[4]^. Laparoscopic surgery enables direct visualization of the pelvic organs and the collection of tissue samples for biopsy^[3]^. Overall, a precise diagnosis and appropriate management of the condition may require a combination of clinical evaluation, imaging investigations, and surgical intervention. There is an urgent need for non-invasive methods to detect endometriosis early, which has led to significant efforts in developing biomarkers in this research field^[5,6]^. Despite comprehensive research conducted over the last twenty years, disease-specific biomarkers for diagnosing and/or staging endometriosis remain unidentified and unvalidated in multisite clinical studies^[7]^.

The analysis of peritoneal fluid, which surrounds endometriotic lesions, may hold the key to a better understanding of endometriosis. Peritoneal fluid plays a crucial role in the development of endometriosis, serving as a dynamic interface between the immune and reproductive systems^[7]^. It is largely an ovarian exudation product that results from increased vascular permeability, with cyclical volume changes and steroid hormone concentrations that are always higher than those in plasma^[7,8]^. Numerous studies have shown that the number and activity of various immune cells in the peritoneal fluid of women with endometriosis, as well as the expression levels of secreted cytokines and inflammatory mediators, are altered^[9]^. Recent research has revealed the presence of approximately 40 distinct immune cell types inside the peritoneal cavity^[10]^. The composition of peritoneal fluid appears to be affected by the release of follicular fluid during ovulation^[11]^.

Extracellular vesicles (EVs) are proposed as possible biomarkers for endometriosis because they transport multiple molecules associated with this condition^[5]^. Research has explored the therapeutic potential of EVs in endometriosis. Studies indicate that EVs derived from peritoneal macrophages can suppress angiogenesis, migration, and invasion in mouse models of the disease^[12,13]^. Additionally, EV-associated miR-214 has been investigated for its role in reducing fibrosis^[14]^, while miR-301a-3p, found in elevated levels within endometriosis lesions, has been linked to macrophage activity modulation when downregulated^[15]^.

Recent advances in understanding EVs in endometriosis reveal distinct differences between EVs released from the endometrium of affected and unaffected women^[16]^. EVs derived from human primary endometrial stromal cells exhibit autocrine and paracrine effects that may influence disease progression, particularly through angiogenesis^[17]^. Additionally, our previous investigation into platelet activation in endometriosis found a significantly higher proportion of small EVs in peritoneal fluid testing positive for platelet biomarkers, suggesting a potential role in disease pathology^[18]^.

In the field of EV research, Raman spectroscopy and atomic force microscopy (AFM) are especially advantageous for their capacity to deliver comprehensive molecular and structural insights without the need for substantial sample preparation^[19]^. Raman spectroscopy is a non-invasive method that allows the identification and analysis of diverse biomolecular constituents within EVs, including proteins, lipids, and nucleic acids, therefore providing insights into their composition and potential functions in biological processes^[20]^. AFM enables the assessment of the combined morphological and nanomechanical properties of individual particles in biological samples, and is explicitly recognized by the Minimal Information for Studies of Extracellular Vesicles (MISEV) 2024 guidelines as a valid orthogonal imaging method for EV morphological characterization^[21]^.

In this study, our primary focus was the comparison between peritoneal fluid-derived EVs from patients with endometriosis and those from control subjects to identify characteristics associated with the pathology that go beyond the expression of single molecular markers. Secondly, we conducted biochemical and biophysical analyses to highlight the properties of small extracellular vesicles (sEVs) derived from peritoneal fluid and follicular fluid to verify the potential impact of follicular fluid on peritoneal fluid EV composition. In fact, although both peritoneal fluid and follicular fluid have a common ovarian origin, their physiological roles are different, as evidenced by the fact that follicular fluid naturally induces the acrosomal response of sperm, while peritoneal fluid does not^[22]^. The disparity in progesterone levels may be a crucial determinant of these fluids’ capacity to stimulate sperm acrosome reaction^[22]^. Understanding these distinctions may have significant implications for fertility therapies and reproductive health.

## METHODS

### Sample information of the cohort analyzed in the study

Patients who underwent elective laparoscopy for suspected endometriosis due to pain complaints and/or infertility were recruited during the preoperative evaluation. After receiving permission from the Institutional Review Board (Protocol IRB-BURLO 07/2023, 31.08.2023), patients were asked to complete an informed consent form. The patient cohort [Table 1] was described in a previous study^[18]^. The study included two peritoneal fluid groups: women with surgically confirmed endometriosis (endometriosis group, *n* = 7) and women without endometriosis (control group, *n* = 7). The control group consisted of patients who underwent laparoscopy for benign, non-endometriotic conditions, in whom the absence of endometriosis was verified by direct surgical and visual inspection of the pelvic cavity. Obtaining peritoneal fluid from asymptomatic, healthy women is ethically and clinically unfeasible, as it would require an invasive laparoscopic procedure without clinical indication. Thus, this control group represents the closest ethically permissible comparator and mirrors the design adopted by previous studies on peritoneal fluid EVs in endometriosis^[18]^. Three patients who underwent assisted reproductive technology (ART) were also enrolled. During the oocyte retrieval procedure (transvaginal follicular aspiration), follicular fluid was collected from the first and largest punctured follicle in each ovary. Each ovarian follicle underwent independent aspiration to ensure precise collection. Follicular fluid sEVs were included as an intra-patient, inter-compartment biological reference to contextualize EV composition across distinct pelvic fluid environments, given that follicular fluid released at ovulation can contribute to the peritoneal fluid milieu. Their inclusion was not intended to identify endometriosis-specific biomarkers, but to characterize the molecular baseline of sEVs from a distinct, physiologically related compartment within the same patient cohort.

**Table 1:** Peritoneal fluid collection: clinical characteristics

**Patient**	**Symptoms/medical history**
Control 1	Leiomyomatosis
Control 2	Uterine fibromatosis
Control 3	Leiomyomatosis
Control 4	Myomatosis
Control 5	Uterine fibromatosis
Control 6	Uterine fibromatosis
Control 7	Uterine fibromatosis
Endometriosis 1	Paucisymptomatic. Vitiligo, Atopic dermatitis. II ASRM Leiomyomatosis
Endometriosis 2	Leiomyomatosis, Adenomyosis, Hashimoto, Celiac Disease
Endometriosis 3	Dysmenorrhoea, Dyspareunia, Chronic pelvic pain, Catamenial dyschezia. No comorbidities. IV ASRM
Endometriosis 4	Paucisymptomatic. No comorbidities. IV ASRM
Endometriosis 5	Dysmenorrhoea, Dyspareunia. No comorbidities. II ASRM. Ovarian Leiomyoma
Endometriosis 6	Dysmenorrhoea, Dyspareunia, Chronic pelvic pain, Catamenial dyschezia. No comorbidities. I ASRM, Adenomyosis, Leiomyomatosis
Endometriosis 7	Dysmenorrhoea, Dyspareunia. Pelvic Varicocele, No other comorbidities

ASRM: American Society for Reproductive Medicine.

### Single-particle interferometric reflectance imaging sensing: exoview R100 analysis

The investigation was performed via single-particle interferometric reflectance imaging sensing (SP-IRIS) via the ExoView R100 system and the ExoView Human Tetraspanin Kit (NanoView Biosciences, Brighton, MA, USA) as previously described^[23]^. This antibody-based single-particle capture and detection assay simultaneously quantifies EV-positive surface markers (CD9, CD63, CD81) at the single-particle level, fulfilling the MISEV 2024 requirement for EV-positive marker characterization. The platform further enables the identification of tetraspanin co-expression patterns, providing single-particle immunophenotyping data that goes beyond bulk ensemble measurements.

### Isolation of EVs

EVs were isolated from the peritoneal fluid and follicular fluid samples of the enrolled subjects by size exclusion chromatography (SEC) (qEV/70 nm Gen2; Izon Science, Christ-church, New Zealand). 500 μL of each sample was loaded in the SEC column, and fractions 2-7 (400 μL each) were collected.

EV samples were characterized by Nanoparticle Tracking Analysis (NTA) (NanoSight NS300; Malvern, Panalytical LTD, Malvern, UK) to evaluate their size distribution and concentrations. For the analysis, samples were diluted in fresh filtered phosphate buffered saline (PBS) and injected into the sample chamber through a syringe pump that provides a continuous flow of new particles into the sample chamber. Recordings of the particle movements were collected for 60 s, 3 times for each sample.

### AFM

AFM images of EVs were captured using a method detailed elsewhere^[21,24]^. Images were taken in PeakForce mode on a Multimode 8 microscope (Bruker, USA) equipped with Scanasyst Fluid+ probes (Bruker), a Nanoscope V controller, a JV-type piezoelectric scanner, and a sealed fluid cell. Briefly, 5 μL aliquots from EV samples were placed on poly-L-lysine-coated glass coverslips and allowed to adsorb for 30 min at 4 °C before being inserted directly into the fluid cell without drying. The sample concentration was adjusted through successive depositions to optimize the number of isolated particles. Quantitative morphometric analysis was conducted using Gwyddion 2.61^[25]^ and custom Python scripts. For each particle, two parameters were measured: spherical diameter in solution (D) and the contact angle (CA) upon adsorption. The CA has been shown to correlate directly with the mechanical stiffness of intact vesicles.All statistical analyses were performed using OriginPro (Version 2023b and 2025, OriginLab Corporation, Northampton, MA, USA).

### Raman spectroscopy

Before the Raman analysis, the EV samples from peritoneal fluid of women with endometriosis (*n* = 7), women without endometriosis who underwent surgery for benign, noninflammatory conditions (*n* = 7), and follicular fluid samples (*n* = 3) obtained by SEC were concentrated by ultracentrifugation (100,000 × *g* for 70 min at 4 °C; L7-65; Rotor SW60; Beckman Coulter, Brea, CA, USA). NTA analysis was repeated to verify the particle concentration prior to proceeding with the spectroscopic analysis.

The Raman analysis of sEV samples was performed as previously described^[26]^. Briefly, a 4 μL drop of sEVs was dried at room temperature on a CaF_2_ disk, and the Raman spectra were collected using the Raman microscope Aramis (Horiba Jobin-127 Yvon, France) coupled to a laser source at 532 nm and a 50× objective (Olympus, Tokyo, Japan). For all the analysis, an acquisition time of 30 s for two accumulations, a diffraction grating at 1,800 grooves/mm, a 400 μm entrance slit, and a 600 μm slit were used. Spectra were acquired using LabSpec6 software (Horiba Scientific) in the spectral ranges 500-1,800 cm^-1^ and 2,700-3,200 cm^-1^ with a spectral resolution < 1.2 cm^-1^. For all samples, about 10 spectra/drop were acquired at random points at the edges of the drop area.

After the Raman data acquisition, spectra were fit with a polynomial baseline, resized on the reference band at 1,004 cm^-1^, and normalized through unit vector normalization in order to compensate for autofluorescence and background interference. Raman spectra were analyzed by descriptive and multivariate statistical analysis using OriginPro (Version 2023b and 2025), as previously described^[26]^. Average Raman spectra were obtained for follicular-derived sEVs, endometriosis peritoneal sEVs, and control peritoneal sEVs.

Classical least squares (CLS) was used to quantify the spectral contribution of reference molecules in the average spectrum of sEV. Briefly,, phosphatidylcholine (16:0/22:6; PC), phosphatidic acid (PA), and phosphatidylserine (PS) were purchased from Avanti Polar Lipids (Alabaster, AL, USA) and Raman spectra were obtained using the same acquisition settings used for sEV. The PC, PA, and PS spectra were used as reference loading for CLS fitting in Labspec 6 software. The resulting normalized scores described the relationships between the considered lipids and the sEV spectra.

## RESULTS

### Tetraspanin profile

The profile of sEVs from follicular fluid was consistent with previous reports^[23]^, showing that the CD9+ population was the least prevalent, whereas the CD63+ and CD81+ populations were more prevalent. This profile differed from that of peritoneal fluid sEVs, in which CD63+ was the least abundant, while CD81+ and CD9+ were more abundant and present at comparable levels [Figure 1].

**Figure 1:**
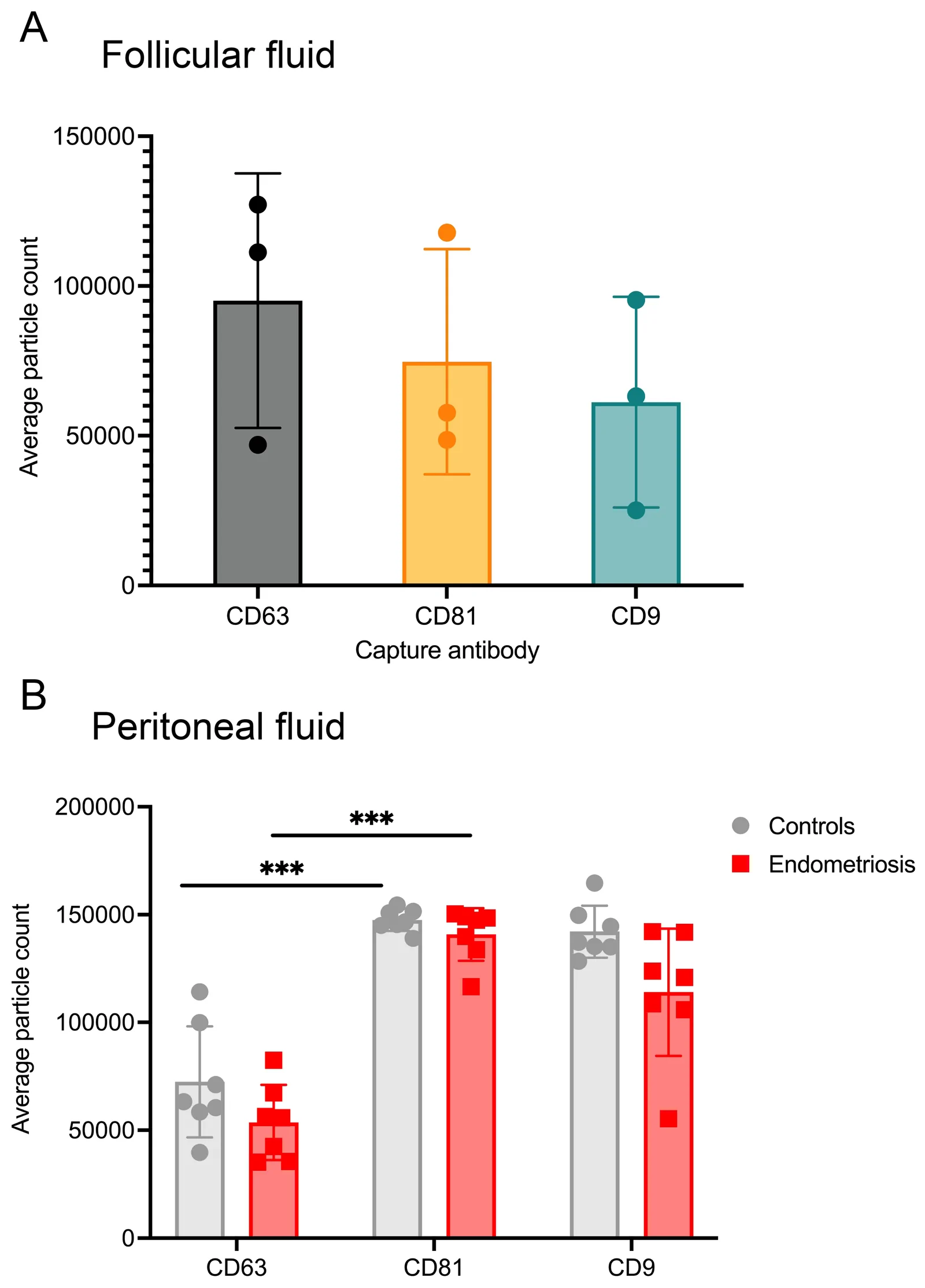
Characterization of tetraspanin profile in sEVs. sEVs were isolated from (A) follicular fluid (*n* = 3), revealing a distinct feature where the CD9+ population is the least abundant; (B) peritoneal fluid of endometriosis patients (*n* = 7) and controls (*n* = 7). The tetraspanin profile in sEVs derived from endometriosis patients was similar to that of controls; specifically, CD81+ and CD9+ populations were more prevalent, whereas the CD63+ population was less abundant. Bars indicate mean ± 1 standard deviation; dots and squares represent individual samples. Data comparisons were performed between indicated groups using the non-parametric Mann-Whitney *U* tests. No multiple-comparison correction was applied. ^***^P < 0.001 after Mann-Whitney *U* test. sEVs: Small extracellular vesicles.

### AFM

Single-particle AFM morphometry was conducted on 13 sEV samples enriched from peritoneal fluid of endometriosis patients (*n* = 5), control subjects (*n* = 5), and follicular fluids (*n* = 3). All samples contained large quantities of particles consistent with the morphological and mechanical characteristics of intact EVs^[21,24]^. As individual samples of the same type were indistinguishable, data from sEVs from the same source were pooled to enhance clarity of presentation.Vesicle diameter (D) and CA distributions were obtained via the morphometrical measurement of 3,294 individual particles found in endometriosis patients’ samples, 2,258 found in control samples, and 3,780 in follicular fluid samples; results are summarized in Figure 2.

**Figure 2:**
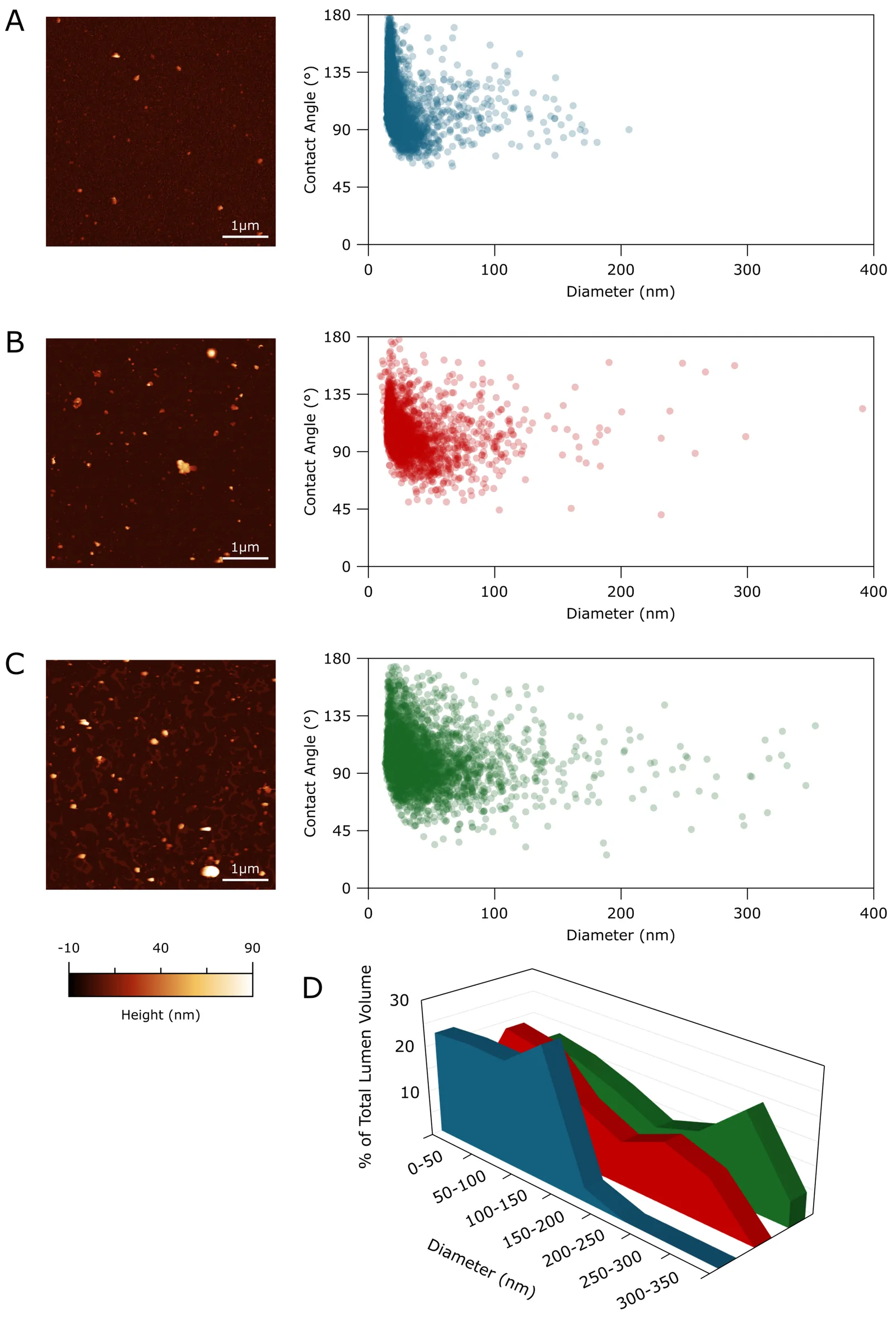
(A-C) Representative 5 µm × 5 µm AFM micrographs (left) and single-particle AFM morphometry plots (right) of sEVs isolated from (A) follicular fluids, (B) peritoneal fluids of control subjects, and (C) peritoneal fluids of endometriosis patients. Each dot in the morphometry plots corresponds to an individual particle plotted according to its free diameter in solution (nm) and surface CA, the latter reflecting its mechanical stiffness, and (D) Percentage of total particle lumen volume *vs*. vesicle diameter as estimated via AFM morphometry. Blue: follicular fluids, red: peritoneal fluids of control subjects, green: peritoneal fluids of endometriosis patients. The entirety of the lumen volume found in particles isolated from follicular fluids is enclosed by EVs with diameters below 200 nm, whereas in both peritoneal fluid samples, roughly half of the lumen volume is enclosed by vesicles with diameters in the 200-400 nm range. AFM: Atomic force microscopy; EVs: extracellular vesicles; sEVs: small extracellular vesicles.

Across all three sample types, the average CA of sEVs was consistent with values observed for intact EVs in the same experimental conditions^[24]^. The average stiffness of sEVs from endometriosis patients (CA = 103° ± 22°) and control subjects (CA = 105° ± 19°) was very similar, whereas sEVs from follicular fluids were marginally stiffer (CA = 112° ± 21°; *P* < 0.0001 for equality of medians with peritoneal‑fluid samples, as assessed by a Wilcoxon rank‑sum test at the 0.05 significance level).

Vesicles enriched from follicular fluid (FF-sEVs) also found to be significantly in size distribution from peritoneal fluid‑derived sEVs. Specifically, 99% of FF‑sEVs had diameters below 100 nm, whereas the 99th percentile was 200 nm for endometriosis‑derived EVs and 160 nm for control EVs. Because size distributions in EV isolates are typically log-normal^[27]^, arithmetic means and standard deviations are inappropriate descriptors. However, AFM morphometry allows to calculate the volume of individual particles in a distribution, offering an alternative method of visualizing significant differences in EV size distributions. Using these calculations, we found that only 3% of the total lumen volume of FF‑sEVs derived from particles with diameters > 150 nm. In contrast, 50% of the total lumen volume in samples enriched from both types of peritoneal fluids derived from vesicles in the 150-350 nm diameter range. These results are summarized in Figure 2D; the distribution of particle lumen volumes in follicular‑fluid samples appears more skewed toward smaller vesicle sizes and differs significantly from those of both peritoneal‑fluid groups (*P* < 0.0001), according to a Kruskal-Wallis test at the 0.05 significance level.

Together, these AFM findings indicate that FF-sEVs exhibit significant differences in size and mechanical stiffness compared to both peritoneal fluids sample types, which are largely comparable to each other.

### Raman spectroscopy

The Raman analysis was successfully performed on all sEV samples from follicular and peritoneal fluids in the spectral ranges 600-1,800 cm^-1^ and 2,600-3,200 cm^-1^. All samples exhibited a favorable signal to noise ratio and the expected protein-related peaks and bands [Amide I (1,600-1,690 cm^-1^) and Amide III (1,200-1,300 cm^-1^)] and nucleic acid-related bands (720-820 cm^-1^), with dominant lipid-related bands in the 2,600-3,200 cm^-1^ range. [Figure 3A].

**Figure 3:**
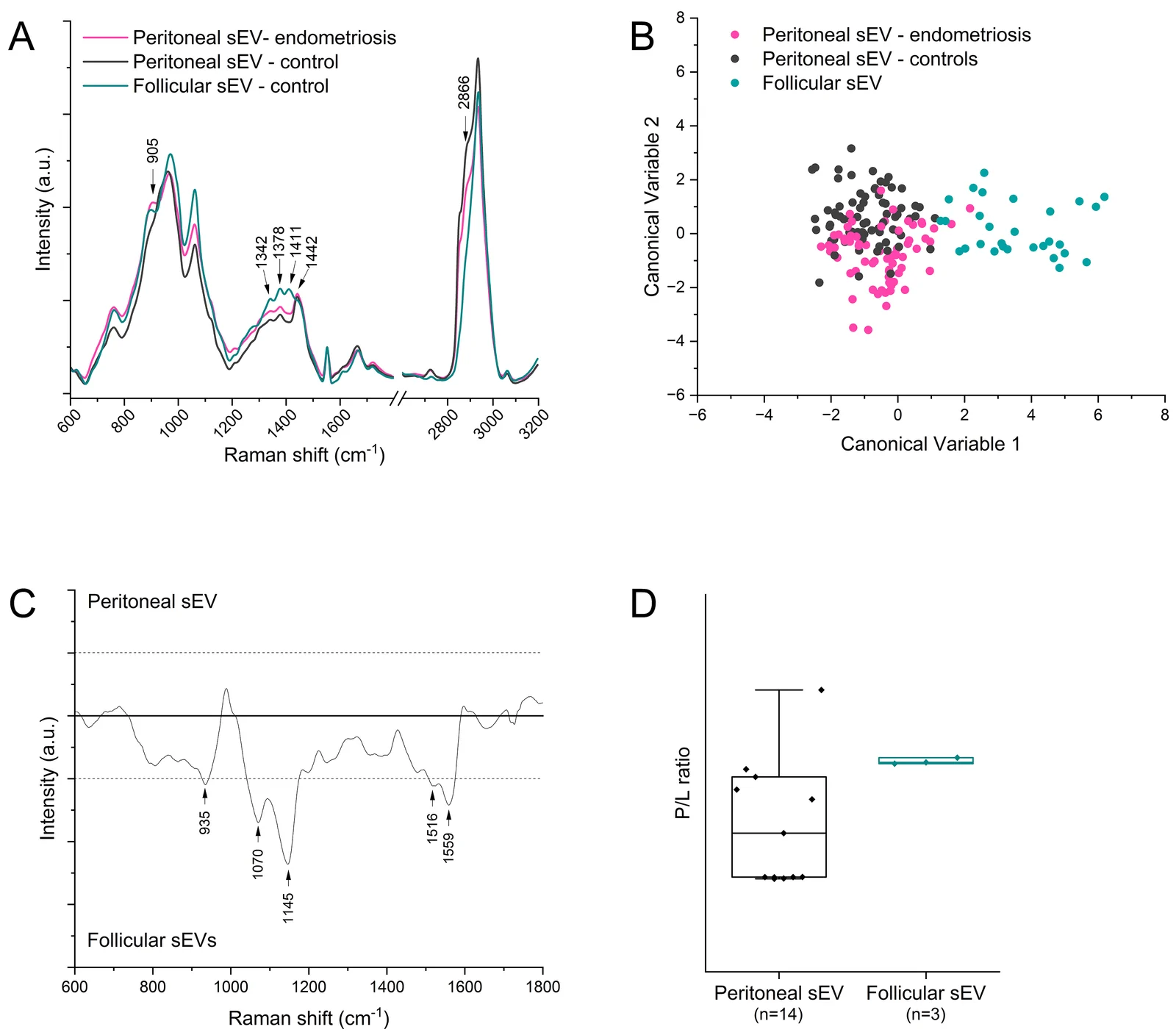
(A) Average Raman spectra obtained from samples of sEVs isolated from the peritoneal fluid of controls (grey line), peritoneal fluid of women with a diagnosis of endometriosis (magenta line), and follicular fluid (green line). Arrows highlight those peaks that change most between the considered groups; (B) Scatter plot of the canonical variable scores obtained after the PCA-LDA analysis of the considered samples. Each dot represents one single spectrum of sEVs; (C) Subtraction spectrum obtained by the subtraction of the average Raman spectrum of follicular sEVs from the Raman spectrum of peritoneal sEVs. Peaks that significantly differ between the two samples (intensity > 0.05) are highlighted with black arrows; and (D) Spectral P/L ratio calculated on the average spectra of sEVs. Each dot represents one single patient. P/L: Protein-to-lipid; PCA-LDA: principal component analysis-linear discriminant analysis; sEVs: small extracellular vesicles.

Although the average spectra largely overlapped, comparison of the three experimental groups revealed significant differences in the Raman signature of follicular fluid‑derived EVs compared with peritoneal fluid‑derived EVs. As shown by the arrows in Figure 3A, the sEV isolated from the follicular fluid showed differences in the presence and intensity of peaks associated with nucleic acids and saccharides (1,378 cm^-1^), proteins (1,411 cm^-1^), and lipids (2,866 cm^-1^) compared to the EVs from peritoneal fluids. On the contrary, a peak between 897 and 905 cm^-1^ was observed in the EVs from follicular fluids and from peritoneal fluid of endometriosis subjects, whereas it was not detectable in the control group. Functional chemical groups were assigned based on previously published findings^[15]^.

To further investigate the reasons for the observed spectral differences, multivariate statistical analysis was performed. The Principal Component Analysis-Linear Discriminant Analysis (PCA-LDA) multivariate analysis was applied to all the collected spectra to verify the ability of the Raman characterization analysis to discriminate between the EV sources (peritoneal *vs*. follicular fluid) as well as between the endometriosis and control groups. As shown in Figure 3B, the model built for the supervised classification of the spectra allowed distinguishing the EVs from peritoneal fluid from those from follicular fluid with an accuracy of 97.7%. Despite the imbalance between the number of samples per category (14 peritoneal fluid-derived EV samples and 3 follicular fluid-derived EV samples), this observation demonstrates a significant difference in the biochemical composition of EVs from the two biofluids that might be associated with the cells of origin, EV cargo or biomolecular corona of the isolated particles. Indeed, these data are in agreement with the AFM results that identify significantly different EV populations in the two biofluids.

Looking more in-depth into the biochemical differences between follicular- and peritoneal-derived EVs, a subtraction spectrum was obtained by the subtraction of the Raman spectrum of follicular-derived sEV from the average spectrum of peritoneal-derived sEV control samples. As shown in Figure 3C, peritoneal and follicular-derived EVs differ in multiple ranges of the spectrum that might account for different macromolecules inside or outside the EVs. Besides, the protein-to-lipid ratio was calculated as it is expected to be modified in EVs based on their size and curvature. Data show a significant difference between peritoneal and follicular sEV, demonstrating that follicular sEVs comprise a population of particles that might be present within the peritoneal-derived sEV but is probably only a minor component of the peritoneal population [Figure 3D].

Focusing on the main aim of our work, the biochemical differences between peritoneal sEVs from control subjects and patients with a diagnosis of endometriosis were investigated. The Raman analysis demonstrates substantial biochemical differences in the considered cohort. The first three principal components obtained by PCA analysis were shown to describe 90.8% of the total variance between the two groups. Indeed, despite similarities possibly due to the biochemical signature of the cells releasing the sEVs within the peritoneal fluid, the loadings of PC2 (16.23% of total variance) and PC3 (7.25% of total variance) highlight some spectral regions that are primarily responsible for the discrimination between the two groups. As shown in Figure 4A, the peaks that account for prominent variations in the Raman spectra of peritoneal sEV between controls and women with endometriosis are those at 891 and 968 cm^-1^, attributable to proteins and lipids (CH wagging), respectively^[28]^. After LDA, the canonical variable scores demonstrated that the spectral signatures of sEV are significantly different between the two experimental groups [Figure 4B] with the possibility to discriminate between controls and women with endometriosis by the sEV Raman fingerprint with a spectrum-level classification accuracy of 68.8%, which, while above chance level, underscores the need for further optimization and validation. Although it is worth noting that the limited sample size and class imbalance warrant caution in interpreting the data before drawing any misleading conclusions, the Mann-Whitney test performed on the canonical variable scores proved that the two groups are significantly different (*P* < 0.001).

**Figure 4:**
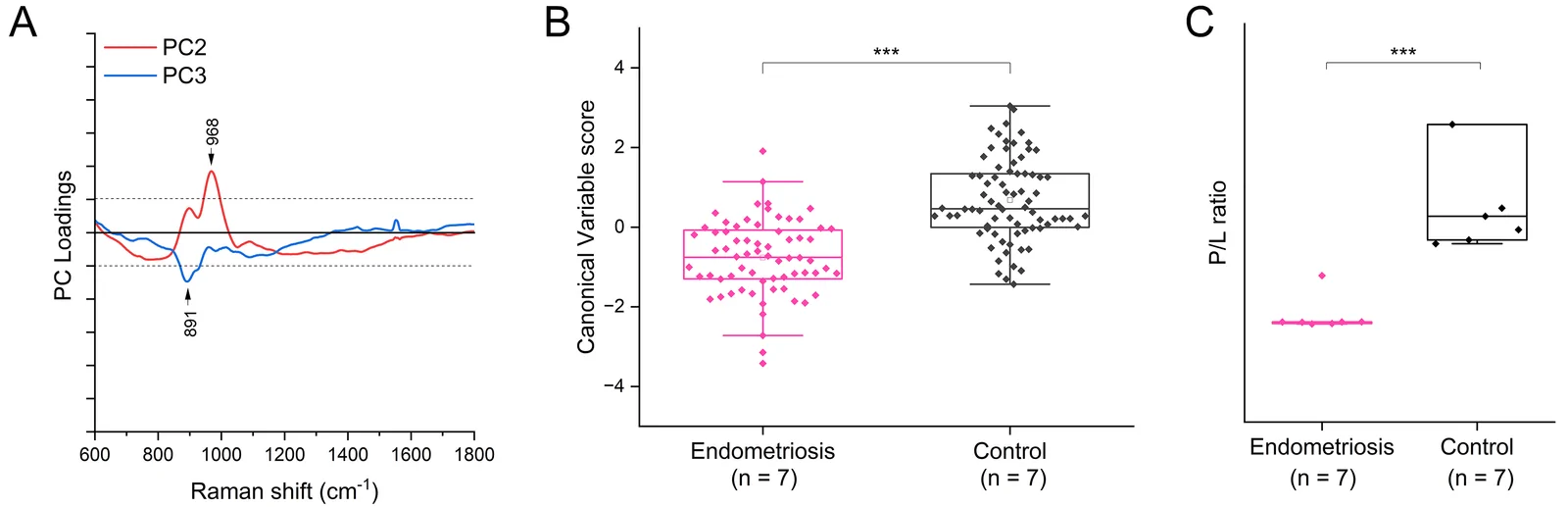
(A) Loadings of PC 2 and 3 obtained after PCA applied to all the spectra from peritoneal sEVs of controls and women with endometriosis. Arrows highlight those peaks that change most, with absolute loading values > 0.05, between the considered groups; (B) Box plot of the canonical variable scores obtained after the PCA-LDA of the considered samples. Each dot represents one single spectrum of peritoneal sEVs, not a single patient. ^***^P < 0.001 after non-parametric Mann-Whitney test applied to canonical variable scores obtained at the spectrum level; note that this analysis was performed at the spectrum level to assess spectral consistency, and statistical significance should be interpreted with caution given the non-independence of multiple spectra per patient; and (C) Spectral P/L ratio calculated on the average spectra of peritoneal sEVs. Each dot represents one patient. ^***^P < 0.001 after non-parametric Mann-Whitney test. P/L: Protein-to-lipid; PC: principal component; PCA: principal component analysis; PCA-LDA: principal component analysis-linear discriminant analysis; sEVs: small extracellular vesicles.

The spectroscopic protein-to-lipid ratio was calculated on the average spectrum obtained for each subject. As shown in Figure 4C, the values obtained for the two groups demonstrate that the biochemistry of peritoneal sEV is significantly different in women with endometriosis compared to controls with a reduced amount of protein per lipid in endometriosis samples (*P* < 0.001 after Mann-Whitney test). Although these data require further validation of a wider cohort of subjects, it is worth noting that the endometriosis samples show a very low variability in the spectroscopic protein-to-lipid ratio, whereas much higher variability is observed in the control group.

These data demonstrate that sEVs from peritoneal fluid exhibit significant biochemical modifications in the context of endometriosis, suggesting that these differences may reflect disease-associated pathophysiological changes. Further studies in larger, well-controlled cohorts are needed to evaluate whether these molecular features could support future biomarker development.

To further investigate the lipid composition of peritoneal sEVs and to contextualize the Raman findings with previously reported lipidomics data^[29]^, the average spectra of peritoneal sEVs were tested for the contribution of specific lipids using CLS fitting analysis. After the acquisition of standard molecules of PS, PC, and PA in the same experimental setting of sEV, the spectral contribution of these molecules to the sEV signature was calculated. Figure 5 shows the normalized scores of the three considered components that highlight differences in the lipid composition of peritoneal and follicular sEV. Specifically, comparing control samples of peritoneal and follicular sEV, remarkable variation in the levels of the considered lipids was observed, with an increased contribution of PC and the detectable contribution of PS in peritoneal sEV compared to follicular sEV.

**Figure 5:**
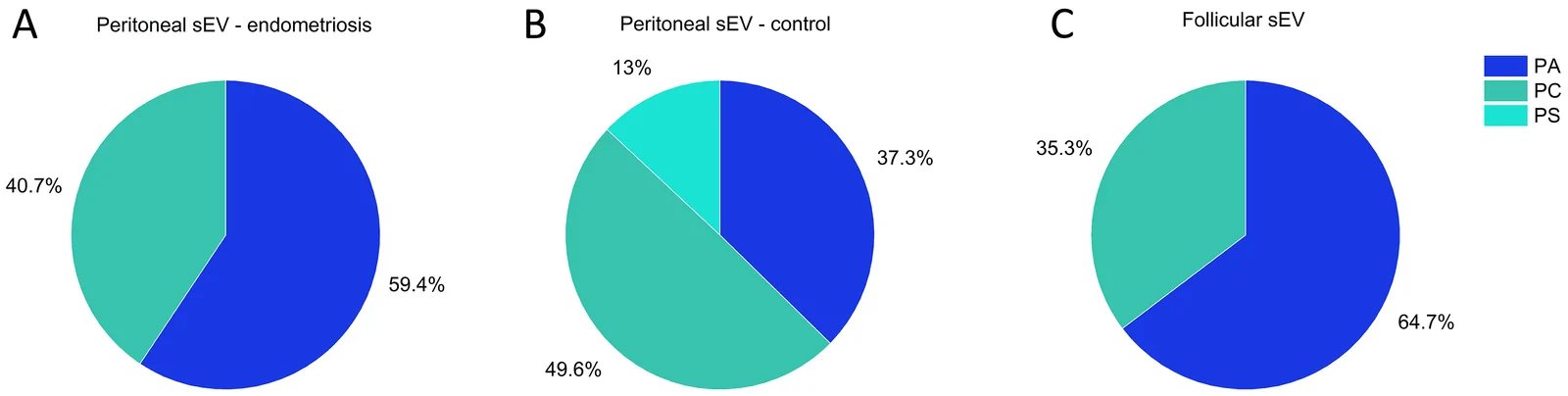
CLS fitting results obtained by fitting the average Raman spectra of sEVs using the reference spectra of PC, PA, and PS as basis components, with normalized scores expressed as fractional contributions to the total lipid signal. Panels show results for (A) Peritoneal fluid, control subjects (without endometriosis); (B) Peritoneal fluid, women with endometriosis; and (C) Follicular fluid, control subjects. CLS: Classical least squares; PA: phosphatidic acid; PC: phosphatidylcholine; PS: phosphatidylserine; sEVs: small extracellular vesicles.

The comparison of peritoneal sEV from control women and women with endometriosis suggested a decrease of PC and PS spectral contribution in the endometriosis group, with PS that becomes undetectable [Figure 5]. On the contrary, PA spectral contribution seems to increase significantly, from 37.3% in the control group, to 59.4% in the endometriosis group.

## DISCUSSION

Peritoneal fluid constitutes the milieu for the fallopian tubes, oocytes, and sperm, potentially influencing the regulation of normal reproductive functions^[30]^.

A small amount of serous fluid in the Pouch of Douglas is normal and helps facilitate the smooth movement of pelvic organs. However, the composition of this fluid is influenced by various factors, which can themselves impact reproductive processes^[22]^. The physiological factors that can lead to the accumulation of free fluid in the pelvis include the following: (i) Menstrual fluid - during menstruation, small amounts of menstrual fluid may seep into the Pouch of Douglas in women of reproductive age; (ii) Ovulation - the rupture of the ovarian follicle during ovulation can release blood or serous fluid into the pelvic cavity; (iii) Peritoneal fluid - small amounts of peritoneal fluid typically gather in the pelvic cavity, with some potentially entering the Pouch of Douglas.

Consistent with our previous analyses of fluid from the Pouch of Douglas^[18]^, we excluded samples with blood contamination to prevent data misinterpretation and rule out menstrual blood contamination. In this study, sEVs isolated from peritoneal fluid and follicular fluid exhibit distinct characteristics. Specifically, sEVs from follicular fluid were significantly smaller in size and had a slightly higher mechanical stiffness. This increased rigidity may reflect a protective mechanism for preserving their contents during transport or for enhancing their interaction with target cells. This finding aligns with Raman characterization data, which revealed significant spectroscopic differences in the molecular fingerprint of these sEVs compared to peritoneal fluid. The high protein-to-lipid ratio in the analyzed particles, possibly due to their small size, and variations in lipid composition were also noted. However, in this small cohort, no correlation was found between rigidity levels and spectroscopic features. Collectively, our findings indicate a potential impact of ovulation on EV composition and emphasize the importance of considering the menstrual cycle phase when studying peritoneal fluid EVs. Further research is necessary to explore the implications of these differences in EV composition for understanding the role of EVs in the peritoneal environment throughout different menstrual cycle phases.

Secondly, this study shows that sEVs isolated from the peritoneal fluid of women with endometriosis and those without endometriosis, who had surgery for benign, non-inflammatory conditions, display significant differences in their Raman spectra. These results should be interpreted as exploratory rather than as evidence of diagnostic readiness. Parlatan *et al*. have previously used Raman spectroscopy combined with PCA and classification algorithms as a non-invasive method to diagnose endometriosis using blood serum^[31]^. Their initial findings suggested that Raman spectroscopy could be a useful non-invasive diagnostic tool for endometriosis, but further research is needed to understand the biological basis of the observed spectral variances. Our data support the hypothesis that Raman spectroscopy of peritoneal sEVs may capture disease-associated biochemical differences, providing a conceptual basis for further investigation in larger and better-controlled cohorts. Furthermore, the lipid composition of EVs isolated from the peritoneal fluid of endometriosis patients, compared to controls, shows changes similar to those seen in the eutopic endometrium in previous studies^[29]^. Specifically, Li *et al*. noted alterations in the levels of PA, PC, and PS in the levels of eutopic endometrium of early-stage endometriosis^[29]^. Specifically, PC and PS levels were decreased, while PA levels were elevated in endometriosis patients. Our data on PA, PC, and PS contents in sEVs isolated from peritoneal fluids corroborate thesepatterns reported in the literature for the eutopic endometrium.

CLS fitting was already proved to be effective in the identification of lipid modification in EVs isolated from different cell sources^[20]^, and to highlight modifications induced by inflammation^[32]^. In the present study, the statistical investigation of the molecular components affecting the Raman signature of sEV confirms that their lipid composition may be influenced by the underlying pathophysiology of endometriosis. Further research is needed to elucidate the specific mechanisms driving these lipid changes and their potential role in the pathogenesis of the disease.

Finally, although the eutopic endometrium was suggested as an ideal specimen for analyzing the lipid profile of endometriosis for identifying potential biomarkers^[29]^, the Raman analysis of peritoneal sEV and the evaluation of their lipid components by Raman spectroscopy represents a cost-effective and automatable approach that, pending validation in larger and well-controlled cohorts, inform future diagnostic or translational research.Several limitations of the present study should be explicitly acknowledged. First, the small sample size (*n* = 7 per group) is the most significant constraint, as it limits statistical power and the generalizability of the findings. The results should therefore be considered exploratory and hypothesis-generating, requiring confirmation in larger, independently recruited cohorts. Second, regarding EV characterization, while particle size and concentration were assessed by NTA, EV-positive surface markers (CD9, CD63, CD81) were profiled by SP-IRIS (ExoView R100), a platform that employs methodologies recommended by MISEV 2024 for single-particle immunophenotyping, and morphological characterization was performed by AFM, which aligns with MISEV 2024 recommendations for orthogonal imaging techniques, several recommended assays were not performed. These include Western blot analysis for EV-associated markers (e.g., TSG101, Alix) and Western blot analsyis of EV-negative contamination markers (e.g., calnexin, GM130) on both EV preparations and raw peritoneal fluid lysates, as well as Transmission Electron Microscopy (TEM) imaging, were not performed. These assays are recommended for full MISEV 2024 compliance. Their absence is primarily due to the limited and non-renewable nature of peritoneal fluid samples, collected under strictly controlled ethical conditions, which precluded retrospective allocation of dedicated aliquots. Future prospective studies should plan for these assays from the outset. Third, the cross-sectional design of the study does not allow inference about longitudinal EV dynamics across disease stages or in response to treatment. Fourth, the study is single-center, which may introduce selection bias related to local patient referral patterns and clinical practice. Additionally, potential confounding variables such as age, body mass index, and other clinical parameters were not systematically controlled for in this small cohort. Finally, the menstrual cycle phase at the time of sample collection was not systematically recorded, which may represent a confounding variable given the known cyclical variation in peritoneal fluid composition.

In conclusion, our findings highlight significant biochemical differences in sEVs from the peritoneal fluid of endometriosis patients compared to controls. Additionally, distinct biochemical and morphometric variations in follicular fluid-derived sEVs suggest unique functional roles in reproductive processes. Notably, lipidic differences in these vesicles may reflect disease progression, influencing cellular signaling, membrane dynamics, and inflammatory responses. While these findings are exploratory and require validation in larger, rigorously controlled cohorts, they provide a foundation for future mechanistic and clinical investigations into the role of sEVs in endometriosis pathophysiology.

## DECLARATIONS

### Availability of data and materials

The original data presented in the study are openly available in Zenodo at https://zenodo.org/records/15719823.

### Copyright

© The Author(s) 2026.
